# Posture Dynamic Modeling and Stability Analysis of a Magnetic Driven Dual-Spin Spherical Capsule Robot

**DOI:** 10.3390/mi12030238

**Published:** 2021-02-26

**Authors:** Huiyuan Yang, Yongshun Zhang, Zhenhu Liu, Xu Liu, Guanxi Liu

**Affiliations:** Key Laboratory for Precision & Non-Traditional Machining of Ministry of Education of China, Dalian University of Technology, Dalian 116023, China; f1912356@mail.dlut.edu.cn (H.Y.); 18713927503@mail.dlut.edu.cn (Z.L.); syliuxu@mail.dlut.edu.cn (X.L.); liugx@mail.dlut.edu.cn (G.L.)

**Keywords:** dual-spin spherical capsule robot (DSCR), posture stability, Floquet–Liapunov theory, periodic variable coefficient, posture adjustment

## Abstract

In order to realize the intervention operation in the unstructured and ample environments such as stomach and colon, a dual-spin spherical capsule robot (DSCR) driven by pure magnetic torque generated by the universal rotating magnetic field (URMF) is proposed. The coupled magnetic torque, the viscoelastic friction torque, and the gravity torque were analyzed. Furthermore, the posture dynamic model describing the electric-magnetic-mechanical-liquid coupling dynamic behavior of the DSCR in the gastrointestinal (GI) tract was established. This model is a second-order periodic variable coefficient dynamics equation, which should be regarded as an extension of the Lagrange case for the dual-spin body system under the fixed-point motion, since the external torques were applied. Based on the Floquet–Lyapunov theory, the stability domain of the DSCR for the asymptotically stable motion and periodic motion were obtained by investigating the influence of the angular velocity of the URMF, the magnetic induction intensity, and the centroid deviation. Research results show that the DSCR can realize three kinds of motion, which are asymptotically stable motion, periodic motion, and chaotic motion, according to the distribution of the system characteristic multipliers. Moreover, the posture stability of the DSCR can be improved by increasing the angular velocity of the URMF and reducing the magnetic induction intensity.

## 1. Introduction

Compared with traditional endoscopy, the wireless capsule endoscopy gastrointestinal examination is safe, comfortable, and non-invasive, and has obvious advantages in the diagnosis of gastrointestinal diseases, especially for small intestine diseases [1,2]. However, the existing capsule endoscopes lack the functions of active locomotion and position control, so it was also called the passive capsule. Its diagnostic and therapeutic effects are not only limited in three-dimensional ample environments such as stomach and colon [3], but also cannot achieve future functions such as drug delivery, biopsy, and minimally invasive surgery [4]. Therefore, it has become an urgent need to extend the scope of diagnosis and treatment of capsule endoscopes to the three-dimensional ample environment and achieve the active control. Taking the built-in micro-motor as the driving source, the researchers have proposed a variety of active capsules such as bionic type [5], screw type [6], leg type [7], propeller type [8], paddle type [9], and so on. Although the micro-motor-driven capsule can achieve many convenient operations, the power capacity and space in the capsule are limited. The external non-contact driven method is more attractive from the aspects of safety and energy supply. The external non-contact driven method of the micro-robot include the acoustic field [10], the light field [11], the electric field [12], and the magnetic field [13]. Among all the methods mentioned above, the magnetic field actuation is the most promising one for in vivo applications, due to the advantages of high tissue penetration, good biocompatibility, and precise multi-degree-of-freedom control [14].

Magnetically driven capsules can usually be divided into magnetic force and magnetic torque drive modes [15]. For the magnetic force mode, the gradient magnetic field generated by the external permanent magnet or coil can apply magnetic attraction force to the capsule embedded with the permanent magnet, so as to realize the active movement of the capsule in the GI tract [16,17]. Although the magnetic force mode has the advantages of a simple working principle and low cost, the precise movement and control is not always possible, since the magnetic force can vary depending on the angle and the distance between the capsule and the external magnet driver [18]. At the same time, this drive mode also exists the problem of pose singularity in certain areas of the working space [19].

The magnetic torque driving method is mainly divided into two types. One is to use the static magnetic torque generated by the gradient magnetic field to realize the rolling locomotion of the capsule [20,21]. The other is to use the dynamic magnetic torque generated by the uniform rotating magnetic field to drive the capsule [22]. For the preceding type, the magnetic force is coupled with the magnetic torque. Therefore, the control flexibility and motion accuracy seem low. For the latter type, since the uniformly rotating magnetic field eliminates the coupling of magnetic force and magnetic torque, and the arbitrary adjustment of the direction, strength, and rotation speed of the magnetic field can be realized [23], it has higher controllability and flexibility. Although the researchers have realized the active movement of the capsule using the uniform rotating magnetic field [24], the accuracy of posture control needs to be further improved. In fact, the accurate posture control can be achieved only by realizing the separation of the capsule posture adjustment and locomotion. Fortunately, the dual-spin body provides the possibility to achieve this goal.

Meanwhile, to ensure the safe and reliable operation in the GI tract, the stability of the capsule robot needs to be studied. For the stability of the dual-spin body, researchers have launched a series of studies. Likins [25] obtained the posture stability region of the dual-spin spacecraft by using the Routh–Hurwitz criterion. Ling Dehai [26] deduced the posture stability criterion of the dual-spin satellite through the Lyapunov method. Han and Zhang [27] derived the free posture dynamics equation of the dual-spin spacecraft and obtained the conditions for posture stability. Aslanov and Yudintsev [28] studied the posture dynamics and control of a free dual-spin gyroscope spacecraft with variable structure. The posture stability of the aforementioned dual-spin bodies was studied by the sign of the real part of the characteristic root of the differential equation or by constructing of the Lyapunov function. However, the above studies all neglected the effect of external torque. In fact, the external torque has significant impact on the posture stability of the dual-spin body [29]. At the same time, the above stability research methods are only suitable for the linear systems with constant coefficients, rather than the periodic system with variable coefficients. Fortunately, the stability of periodic systems with variable coefficients can be studied by the eigenvalues of the system transition matrix based on the Floquet–Lyapunov theory.

To achieve the accurate posture control of the capsule robot, this paper proposes a dual-spin spherical capsule robot (DSCR) driven by pure magnetic torque, which can achieve the separation and conversion of the posture adjustment and rolling locomotion. Considering the actual working conditions in the GI tract, the posture dynamics equation of the DSCR under the action of external torque was established. By using Floquet–Lyapunov theory, the stability of the periodic variable coefficient dynamic system was studied. The influences of the parameters such as the magnetic induction intensity, the angular velocity of the universal rotating magnetic field (URMF), and the centroid deviation to the system stability were analyzed. 

The contributions of this paper includes: (1) A dual-spin structure capsule robot driven by the URMF was proposed, which solves the problem of coupling between magnetic force and magnetic torque of the magnetic-driven capsule robot. (2) The posture dynamics equation of the DSCR under complex torque was established, which expands the research scope of double-spin body. (3) The posture stability domain of the DSCR for the asymptotically stable motion and periodic motion were obtained.

The rest of the paper is organized as follows. The structure and the working principle of the DSCR are introduced in Section 2. The posture dynamic modeling of the DSCR is presented in Section 3. In Section 4, the posture stability of the DSCR is analyzed, then, experiments are conducted for validation in Section 5. Finally, in Section 6, conclusions are drawn.

## 2. System Overview

### 2.1. The Structure of the DSCR

**Figure 1:**
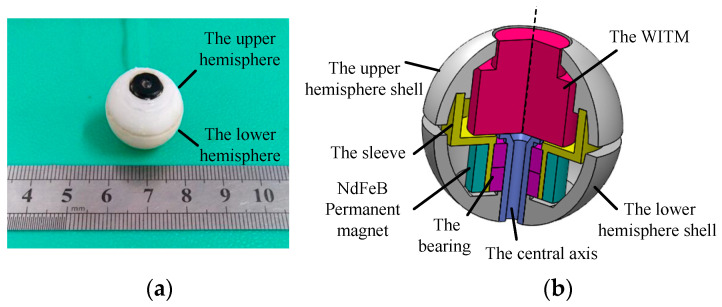
The structure of the dual-spin spherical capsule robot (DSCR). (**a**) The prototype, (**b**) the 3D cross-sectional view.

The prototype and cross-sectional view of the DSCR are shown in Figure 1a,b, respectively. The DSCR is composed of the upper and lower hemispheres, in which the upper hemispherical shell, the sleeve, and the NdFeB permanent magnet are consolidated to form the upper hemisphere. The wireless image transmission module (WITM), the central axis, and the lower hemispherical shell are consolidated to the lower hemisphere. The upper and lower hemispheres are connected by the bearing, and they can rotate relative to each other around the central axis.

The main structural parameters of the DSCR are listed in Table 1. The diameter and weight of the DSCR are 20 mm and 10 g, respectively. The brand of the radially magnetized NdFeB permanent magnet is N50 and the magnetic torque amplitude is 0.2 A.m^2^. The shell of the upper and lower hemispheres can be fabricated by additive manufacturing.

Because the upper hemisphere is fixated with the NdFeB permanent magnet, it can rotate about the central axis under the action of the URMF generated by the tri-axial orthogonal square Helmholtz coils (TOSHC), and the lower hemisphere is under-actuated because of the lack of driving source. Since the upper and lower hemispheres form a coaxial body and have different rotation speed around the center axis, the coaxial body further constitutes a dual-spin body [30]. The rotation of the upper hemisphere makes the DSCR have the attribute of gyroscope, while the under-actuated lower hemisphere provides a stable platform for the WITM.

### 2.2. Three-Phase Current Superposition Formula of the URMF

**Figure 2:**
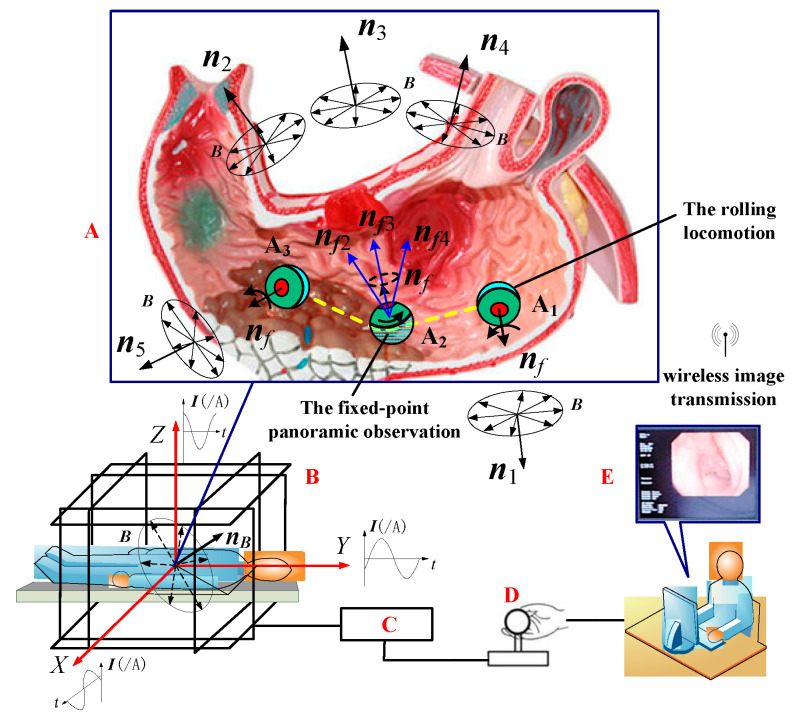
Application scenario of the DSCR and the control system of the universal rotating magnetic field (URMF). A: Double working mode of the DSCR: The fixed-point panoramic observation in the passive mode (***n***_2_, ***n***_3_, ***n***_4_); The rolling locomotion in the active mode (***n***_1_, ***n***_5_). B: The tri-axial orthogonal square Helmholtz coils (TOSHC). C: The URMF controller. D: The Joystick. E: The interactive interface.

As shown in Figure 2, the three-phase alternating current feeding into the TOSHC can generate the URMF after electromagnetic induction and the superposition polarization. The three-phase current superposition formula of the URMF can be expressed as [31]

(1) $$I=\left(\begin{array}{l} I_{x}\\ I_{y}\\ I_{z} \end{array}\right)=\left(\begin{array}{l} I_{0}\sin a\sin(\omega t-\phi_{x})\\ -I_{0}\sin b\sin(\omega t+\phi_{y})\\ I_{0}\sin c\sin(\omega t+\pi /2) \end{array}\right)$$

where, *I*_0_ is the amplitude of the applied electrical current. cosa, cosb, and cosc are the direction cosines of the normal vector ***n_B_*** of the URMF. *ϕ_x_*, *ϕ_y_* are the phase angles, and *ϕ_x_* = arctan (cosc*cosa/cosb), *ϕ_y_* = arctan (cosc*cosb/cosa).

### 2.3. Working Principle of the DSCR

Figure 2 shows the overall medical application scenario of the DSCR inside the stomach. The whole system mainly consists of three parts: (1) the DSCR (A); (2) the TOSHC (B); and (3) the control unit of the URMF (C, D, E).

The implementation scheme is as follows: after the DSCR is swallowed and entered the A_1_ position of the stomach cavity, the doctor adjusts the normal vector of the URMF ***n_B_*** to the horizontal position ***n***_1_ by manipulating the joystick (D) of the controller (C) according to the real-time image transmitted by the WITM. Under the action of the magnetic torque follow-up effect [32], the axis ***n**_f_* of the DSCR can follow ***n***_1_ to reach the horizontal position, and then the DSCR works in the active mode, which can realize rolling locomotion on the surface of the stomach.

When the DSCR reaches the position **A**_2_, ***n_B_*** is adjusted from the horizontal position to the non-horizontal position, and the conversion of the DSCR from the active mode to the passive mode can be realized. When ***n_B_*** is adjusted to the orientations of ***n***_2_, ***n***_3_, and ***n***_4_, the axis ***n**_f_* of the DSCR can be adjusted to ***n**_f_*_2_, ***n**_f_*_3_, ***n**_f_*_4_ in sequence following ***n_B_***. Therefore, the fixed-point panoramic observation can be achieved with the help of the DSCR vision. If the next region needs to be observed, ***n_B_*** can be adjusted again to the horizontal position, as shown by ***n***_5_ in Figure 2. After the DSCR rolls to the position **A**_3_, the above inspection operation process can be repeated.

In summary, the DSCR with the dual-spin structure not only can realize posture control arbitrarily, but can also realize the separation and mutual conversion of the fixed-point posture adjustment and the rolling locomotion.

## 3. Posture Dynamic Modeling

Since the fixed-point posture adjustment function in the passive mode is the key to the conversion of the dual mode, this paper only studies the passive mode of the DSCR.

### 3.1. The Description of the Posture

**Figure 3:**
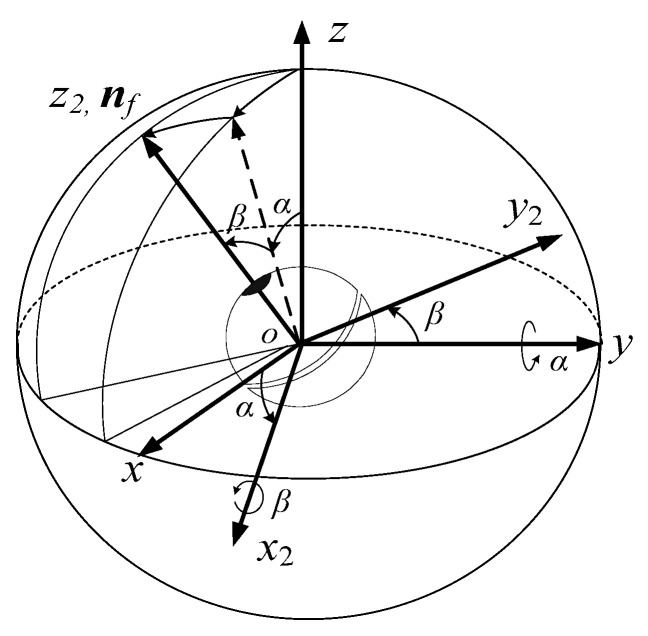
Posture representation of the DSCR.

As shown in Figure 3**,** the posture of the DSCR can be described by the orientation of the *oz*_2_ axis of the coordinate system *ox*_2_*y*_2_*z*_2_, which is connected to the lower hemisphere of the DSCR, and the *oz*_2_ axis is coincident with the axis ***n**_f_*. The coordinate system *ox*_2_*y*_2_*z*_2_ can be obtained by rotating the fixed coordinate system *oxyz* around the *oy* axis by angle *α* (altitude angle), and then around the *ox*_2_ axis by angle *β* (azimuth angle). Since the rotation along the *oz*_2_ axis does not affect the orientation of the DSCR, the posture of the DSCR can be represented by the altitude angle *α* and the azimuth angle *β*, and *ox*_2_*y*_2_*z*_2_ is the résal coordinate system. Considering that the DSCR is an axisymmetric structure and the résal coordinate system *ox*_2_*y*_2_*z*_2_ is the principal axis coordinate system, then the axis ***n**_f_* is the polar axis.

According to Figure 3, the homogeneous transformation matrix ***A***_1_ from the résal coordinate system *ox*_2_*y*_2_*z*_2_ to the fixed coordinate system *oxyz* can be obtained as

(2) $$A_{1}=\left(\begin{array}{ccc} \cos\alpha & \sin\alpha \sin\beta & \sin\alpha \cos\beta \\ 0 & \cos\beta & -\sin\beta \\ -\sin\alpha & \cos\alpha \sin\beta & \cos\alpha \cos\beta \end{array}\right)$$

### 3.2. Torque Analysis

The external torques acting on the DSCR include: the coupling magnetic torque of the URMF and the NdFeB permanent magnet, the viscoelastic friction torque between the DSCR and the GI tract, and the gravity torque introduced by the deviation of the DSCR centroid.

#### 3.2.1. The Coupled Magnetic Torque

**Figure 4:**
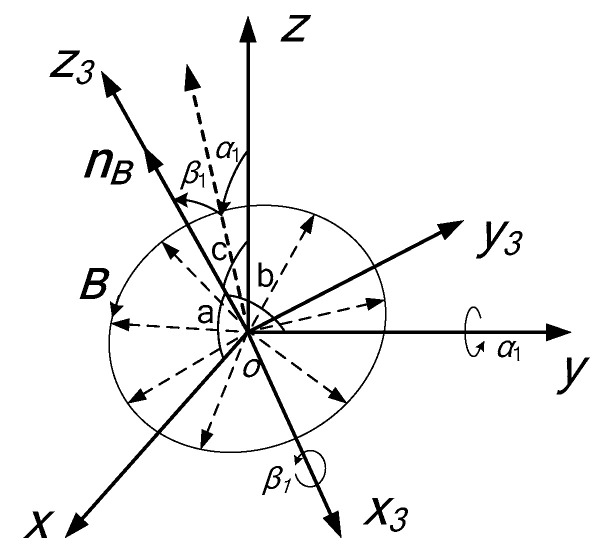
The URMF coordinate system *ox*_3_*y*_3_*z*_3_.

To describe the basic unit of the URMF—the rotating magnetic vector ***B***, the URMF coordinate system *ox*_3_*y*_3_*z*_3_ is introduced with the DSCR spherical center *o* as the coordinate origin. Where, the *oz*_3_ axis coincides with the normal vector of the URMF ***n_B_***, the *ox*_3_, *oy*_3_ axis are located in the rotation plane of the rotating magnetic vector ***B***, and form a right-handed coordinate system with *oz*_3_ axis, as shown in Figure 4.

Similar to the rotation relationship of Figure 3, *ox*_3_*y*_3_*z*_3_ can be obtained by rotating the fixed coordinate system *oxyz* about the *oy* axis by *α*_1_ firstly and then about the *ox*_3_ axis by *β*_1_, then the *oz*_3_ axis can be coincide with ***n_B_***. Therefore, the orientation of the URMF can be expressed by the altitude angle *α*_1_ and the azimuth angle *β*_1_.

According to the Figure 4, the homogeneous transformation matrix ***A***_2_ from the URMF coordinate system *ox*_3_*y*_3_*z*_3_ to the fixed coordinate system *oxyz* can be obtained as

(3) $$A_{2}=\left(\begin{array}{ccc} \cos\alpha_{1} & \sin\alpha_{1}\sin\beta_{1} & \sin\alpha_{1}\cos\beta_{1}\\ 0 & \cos\beta_{1} & -\sin\beta_{1}\\ -\sin\alpha_{1} & \cos\alpha_{1}\sin\beta_{1} & \cos\alpha_{1}\cos\beta_{1} \end{array}\right)$$

The orientation of the URMF can be expressed in the coordinate system *ox*_3_*y*_3_*z*_3_ as

(4) $$n_{B3}={\left(\begin{array}{ccc} 0 & 0 & 1 \end{array}\right)}^{T}$$

The orientation of the URMF can be expressed in the fixed coordinate system *oxyz* as

(5) $$n_{B}={\left(\begin{array}{ccc} \cos a & \cos b & \cos c \end{array}\right)}^{T}$$

where, *a*, *b*, and *c* are the angles between the ***n****_B_* and each coordinate axis of the fixed coordinate system *oxyz*.

Since ***n_B_*** and ***n_B_*_3_** are all the orientations of the URMF, the following relationship are satisfied

(6) $$\begin{array}{l} n_{B}=A_{2}\cdot n_{B3}\\ =\left(\begin{array}{ccc} \cos\alpha_{1} & \sin\alpha_{1}\sin\beta_{1} & \sin\alpha_{1}\cos\beta_{1}\\ 0 & \cos\beta_{1} & -\sin\beta_{1}\\ -\sin\alpha_{1} & \cos\alpha_{1}\sin\beta_{1} & \cos\alpha_{1}\cos\beta_{1} \end{array}\right)\left(\begin{array}{c} 0\\ 0\\ 1 \end{array}\right) \end{array}$$

From the Equation (6), Equation (7) can be derived

(7) $$\alpha_{1}=\arctan\frac{\cos a}{\cos c},\;\beta_{1}=b-\frac{\pi }{2}$$

The rotating magnetic vector ***B*** can be represented in the *ox*_3_*y*_3_*z*_3_ as

(8) $$B_{3}={\left(B\cos\omega t,B\sin\omega t,0\right)}^{T}$$

where, *B* is the magnetic induction intensity of the URMF, and *ω* is the angular velocity of the URMF.

In order to represent the rotating magnetic vector in the résal coordinate system *ox*_2_*y*_2_*z*_2_, ***B*_3_** can be firstly transformed to the fixed coordinate system *oxyz*, then, transformed to the résal system *ox*_2_*y*_2_*z*_2_. Therefore, the rotating magnetic vector can be represented in *ox*_2_*y*_2_*z*_2_ as

(9) $$B_{2}=A_{1}{}^{-1}A_{2}B_{3}=B\left(\begin{array}{l} E_{1}\cos\omega t+E_{2}\sin\omega t\\ E_{3}\cos\omega t+E_{4}\sin\omega t\\ E_{5}\cos\omega t+E_{6}\sin\omega t \end{array}\right)$$

where, the specific forms of *E*_1_, *E*_2_, *E*_3_, *E*_4_, *E*_5_ and *E*_6_ are following as

$$\begin{array}{l} E_{1}=a_{11}\cos\alpha -a_{31}\sin\alpha ,\\ E_{2}=a_{12}\cos\alpha -a_{32}\sin\alpha ,\\ E_{3}=a_{11}\sin\alpha \sin\beta +a_{31}\cos\alpha \sin\beta \\ E_{4}=a_{12}\sin\alpha \sin\beta +a_{22}\cos\beta +a_{32}\cos\alpha \sin\beta ,\\ E_{5}=a_{11}\sin\alpha \cos\beta +a_{31}\cos\alpha \cos\beta ,\\ E_{6}=a_{12}\sin\alpha \cos\beta -a_{22}\sin\beta +a_{32}\cos\alpha \cos\beta \\ a_{11}=\cos\alpha_{1},a_{12}=\sin\alpha_{1}\sin\beta_{1},a_{13}=\sin\alpha_{1}\cos\beta_{1}\\ a_{21}=0,a_{22}=\cos\beta_{1},a_{23}=-\sin\beta_{1}\\ a_{31}=-\sin\alpha_{1},a_{32}=\cos\alpha_{1}\sin\beta_{1},a_{33}=\cos\alpha_{1}\cos\beta_{1} \end{array}$$

Since the symmetrical axis of NdFeB permanent magnet coincides with the polar axis ***n**_f_*, the magnetic dipole moment of the NdFeB magnet can be represented in the *ox*_2_*y*_2_*z*_2_ as

(10) $$m_{2}={\left(m\cos(\omega t-\delta ),m\sin(\omega t-\delta ),0\right)}^{T}$$

where *m* is the magnitude of the magnetic dipole moment. *δ* is the slip angle between the magnetic dipole moment and the rotating magnetic vector.

Based on the magnetic coupling theory [33], the coupling magnetic torque of the URMF and the NdFeB permanent magnet can be expressed in the résal coordinate system *ox*_2_*y*_2_*z*_2_ as

(11) $${\left(\begin{array}{ccc} T_{x2} & T_{y2} & T_{z2} \end{array}\right)}^{T}=m_{2}\times B_{2}={\left(\begin{array}{ccc} C_{1} & C_{2} & C_{3} \end{array}\right)}^{T}$$

where

$$C_{1}=mB\left(E_{5}\cos\omega t\sin(\omega t-\delta )+E_{6}\sin\omega t\sin(\omega t-\delta )\right)\;C_{2}=mB\left(E_{5}\cos\omega t\sin(\omega t-\delta )+E_{6}\sin\omega t\sin(\omega t-\delta )\right)\;C_{3}=mB\left(\begin{array}{l} E_{3}\cos\omega t\cos(\omega t-\delta )+E_{4}\sin\omega t\cos(\omega t-\delta )-\\ E_{1}\cos\omega t\sin(\omega t-\delta )-E_{2}\sin\omega t\sin(\omega t-\delta ) \end{array}\right)$$

#### 3.2.2. The Viscoelastic Friction Torque

**Figure 5:**
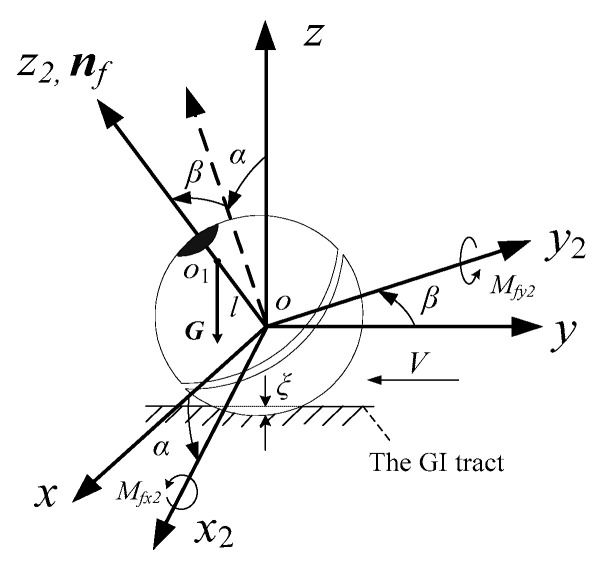
Schematic diagram of the viscoelastic friction torque and the gravity torque.

When the DSCR works in the GI tract, the deformation of the GI tract and the digestive fluid will exert a viscoelastic damping effect on the DSCR, as shown in Figure 5. It can be seen from the literature [34,35] that when the compression deformation *ξ* of the GI tract is small, the rolling speed *V* of the DSCR is much smaller than the speed of sound and the characteristic time *ξ*/*V* is much larger than the dissipation and relaxation time. Then, the torque of the viscoelastic frictional resistance to the sphere center *o* of the DSCR under the quasi-static state can be expressed as

(12) $$M=-RF_{N}k_{0}\omega_{D}$$

where *R* is the radius of the DSCR. *F_N_* is the positive pressure of the DSCR on the contact surface. ***ω****_D_* is the angular velocity of the DSCR. *k*_0_ is the friction coefficient, which can be expressed as [30]

(13) $$k_{0}=\frac{1}{3}\frac{{\left(3\eta_{2}-\eta_{1}\right)}^{2}}{3\eta_{2}+2\eta_{1}}\left[\frac{\left(1-\nu^{2})(1-2\nu \right)}{Y\nu^{2}}\right]$$

where *η*_1_ and *η*_2_ are the viscosity coefficient of the DSCR and the GI tract, respectively. *Y* and *ν* are the Young’s modulus and Poisson’s ratio of the GI tract. This formula relates the friction coefficient to the viscous and elastic constants of the contact material.

From the Equations (12) and (13), the projection of the viscoelastic friction torque in the résal coordinate system *ox*_2_*y*_2_*z*_2_ can be obtained as

(14) $$\{\begin{array}{l} M_{fx_{2}}=-k\dot{\beta }\\ M_{fy2}=-k\dot{\alpha }\cos\beta \\ M_{fz2}=-k\dot{\alpha }\sin\beta \end{array}$$

where *k* is the viscous damping coefficient, $k=k_{0}RF_{{}^{N}}$. $\dot{\alpha }$ and $\dot{\beta }$ are the angular velocity of the DSCR around the *oy* axis and *ox*_2_ axis, respectively.

#### 3.2.3. The Gravity Torque

When the DSCR centroid *o*_1_ moves on the polar axis ***n**_f_*, its own gravity *G* will exert a torque on the sphere center *o*, as shown in Figure 5. The gravity torque can be expressed in the résal coordinate system*ox*_2_*y*_2_*z*_2_ as

(15) $$\left(\begin{array}{c} M_{Gx2}\\ M_{Gy2}\\ M_{Gz2} \end{array}\right)=\bar{oo_{1}}\times G=\left(\begin{array}{c} Gl\cos\alpha \sin\beta \\ Gl\sin\alpha \\ 0 \end{array}\right)$$

where *l* is the modulus of the vector $\bar{oo_{1}}$. When *l* takes a positive value, the centroid is above the sphere center. When *l* takes a negative value, the centroid is below the sphere center. When *l* = 0, the centroid coincides with the sphere center.

According to Equations (11), (14) and (15), the combined external torque acting on the DSCR can be expressed in the résal coordinate system *ox*_2_*y*_2_*z*_2_ as

(16) $$\left\{ \begin{array}{l} M_{x2}=T_{x2}+M_{fx_{2}}+M_{Gx2}\\ M_{y2}=T_{y2}+M_{fy_{2}}+M_{Gy2}\\ M_{z2}=T_{z2}+M_{fz_{2}}+M_{Gz2} \end{array}\right.$$

### 3.3. Posture Dynamics Equation

Based on the theory of angular momentum change of the system in the arbitrary rotating coordinate system [36], the résal coordinate system *ox*_2_*y*_2_*z*_2_ is selected as the rotating coordinate system. The Euler dynamic equation describing the fixed-point posture adjustment of the DSCR can be expressed as

(17) $$\{\begin{array}{l} J_{e}\dot{p}+(J_{1}+J_{2}-J_{e})qr+J_{1}\sigma q=M_{x2}\\ J_{e}\dot{q}-(J_{1}+J_{2}-J_{e})pr-J_{1}\sigma p=M_{y2}\\ J_{1}(\dot{r}+\dot{\sigma })+J_{2}\dot{r}=M_{z2}\\ J_{1}(\dot{r}+\dot{\sigma })=M_{\Delta } \end{array}$$

where *J*_e_ is the equatorial moment of inertia of the DSCR. *J*_1_ and *J*_2_ are the polar inertia moment of the upper and lower hemisphere, respectively. {*p*, *q*, *r*} are the angular velocity of the lower hemisphere in *ox*_2_*y*_2_*z*_2_, and $p=\dot{\beta }$, $q=\dot{\alpha }\cos\beta$, $r=-\dot{\alpha }\sin\beta$. *σ* is the angular velocity of the upper hemisphere relative to the lower hemisphere. *M_x_*_2_, *M_y_*_2_, *M_z_*_2_ are the projections of the external torque in the *ox*_2_*y*_2_*z*_2_. *M*_△_ is the total external torque of the upper hemisphere along the polar axis ***n**_f_* (we assume *M*_△_ = 0) [36]. When the system reaches the steady state, the upper hemisphere rotates synchronously with the URMF, so it can be considered that the constant speed constraint condition is satisfied, that is, *σ* = *ω*. Considering that the resistance torque of the DSCR along the polar axis, ***n**_f_* can be compensated by the driving torque, that is, ***M****_z_*_2_ = 0. Therefore, the last two formulas of Equation (17) can be ignored. 

Summing up, the posture dynamic equation describing the electric-magnetic-mechanical-liquid coupling behaviour of the DSCR in the GI tract can be expressed as Equation (18). Moreover, this equation can be classified as an extension of the Lagrangian case for the coaxial bodies system—the fixed-point motion of the coaxial body under the external recovery/overturning moment.

(18) $$\left\{ \begin{array}{l} J_{e}\ddot{\beta }+J_{1}\omega \dot{\alpha }\cos\beta -(J_{1}+J_{2}-J_{e}){\dot{\alpha }}^{2}\sin\beta \cos\beta =\\ mB\sin(\omega t-\delta )\left(E_{5}\cos\omega t+E_{6}\sin\omega t\right)-k\dot{\beta }+Gl\cos\alpha \sin\beta \\ J_{e}\ddot{\alpha }\cos\beta -J_{e}\dot{\alpha }\sin\beta -J_{1}\omega \dot{\beta }+(J_{1}+J_{2}-J_{e})\dot{\alpha }\dot{\beta }\sin\beta =\\ -mB\cos(\omega t-\delta )\left(E_{5}\cos\omega t+E_{6}\sin\omega t\right)-k\dot{\alpha }\cos\beta +Gl\sin\alpha \end{array}\right.$$

## 4. Posture Stability Analysis

### 4.1. The Floquet–Lyapunov Theory

Since the altitude angle *α* and the azimuth angle *β* are typically small, it can be approximated as sin*ϑ* = *ϑ*, cos*ϑ* = 1, (*ϑ* = *α*, *β*), and the high-order small amount ${\dot{\alpha }}^{2}$, $\dot{\alpha }\dot{\beta }$, $\dot{\alpha }sin\beta$ can be ignored. Introducing the dimensionless time scale *τ* = *ωt*, Equation (18) can be expressed in matrix form as

(19) $$\omega^{2}MX^{\prime \prime }+\omega NX^{\prime }+K(\tau )X=\varepsilon F(\tau )$$

where $X={\left(\alpha ,\beta \right)}^{T}$, $X^{\prime }=dX/d\tau$, *ε* = *mB*, the matrices ***M***, ***N***, ***K***, ***F*** are mass matrix, damping matrix, nonlinear stiffness matrix, and external excitation matrix respectively, and

$$\begin{array}{l} M=\left(\begin{array}{cc} J_{e} & 0\\ 0 & J_{e} \end{array}\right),\;N=\left(\begin{array}{cc} k & -J_{1}\omega \\ J_{1}\omega & k \end{array}\right),\\ K=\left(\begin{array}{cc} K_{11} & K_{12}\\ K_{21} & K_{22} \end{array}\right),F=\left(\begin{array}{c} \left(\sin\alpha_{1}\cos\tau -\cos\alpha_{1}\sin\beta_{1}\sin\tau )\cos(\tau -\delta \right)\\ \left(-\sin\alpha_{1}\cos\tau +\cos\alpha_{1}\sin\beta_{1}\sin\tau )\sin(\tau -\delta \right) \end{array}\right)\\ K_{11}=\varepsilon (\cos\alpha_{1}\cos\tau +\sin\alpha_{1}\sin\beta_{1}\sin\tau )\cos(\tau -\delta )-Gl\\ K_{12}=-\varepsilon \cos\beta_{1}\sin\tau \cos(\tau -\delta )\\ K_{21}=-\varepsilon (\cos\alpha_{1}\cos\tau +\sin\alpha_{1}\sin\beta_{1}\sin\tau )\sin(\tau -\delta )\\ K_{22}=\varepsilon \cos\beta_{1}\sin\tau \sin(\tau -\delta )-Gl \end{array}$$

Since the matrices ***K***(*τ*) and ***F***(*τ*) change periodically with *τ*, Equation (19) is a second-order periodic variable coefficient dynamic equation. Because the stability of the non-homogeneous periodic variable coefficient dynamic equation and the corresponding homogeneous equation have the same necessary and sufficient conditions, the homogeneous form of Equation (19) in the form of first order state variables can be expressed as

(20) $$q^{\prime }=A(\tau )q$$

where $q={\left(X,X^{\prime }\right)}^{T}$, $A=\left(\begin{array}{cc} 0 & I\\ A_{21} & A_{22} \end{array}\right)$, ***0*** represents zero matrix, I is the second-order unit matrix, $A_{21}=-\frac{M^{-1}K}{\omega^{2}}$, $A_{22}=-\frac{M^{-1}N}{\omega }$.

Since the matrices ***A***(*τ*) change periodically with *τ*, Equation (20) is still a periodic variable coefficient dynamic system. According to the Floquet–Lyapunov theory, the stability of the periodic variable coefficient system can be studied according to the eigenvalue λ of its transition matrix ***P*** [37]: If the modulus of all eigenvalues of ***P*** are less than 1, the system is asymptotically stable. If ***P*** has an eigenvalue whose modulus is greater than 1, the system is unstable. If the modulus of the eigenvalues of ***P*** are less than or equal to 1, and at least one of them is equal to 1, the system is limit stable. The eigenvalues of the transition matrix are also called the characteristic multipliers [38]. Therefore, the stability of the dynamic system (20) can be determined by the distribution of the characteristic multipliers of the transition matrix ***P***.

According to the method of C.S.Hu [39], the transition matrix ***P*** of the periodic system can be calculated as

(21) $$\left\{ \begin{array}{l} P=\overset{N_{k}}{\underset{i=1}{\Pi }}\left(I+\sum_{j=1}^{J}\frac{{\left(\Delta_{i}C_{i}\right)}^{j}}{j!}\right)\\ C_{k}=\frac{1}{\Delta_{k}}\int_{\psi_{k-1}}^{\psi_{k}}A(\xi )d\xi ,\;\xi \in \tau_{k} \end{array}\right.$$

where ***I*** is the unit matrix, *N_k_* is the number of parts that divide the period *T* of the periodic system equally, and each average point is represented by *k* = 0, 1, 2, … *N*_k_. The *k*th interval $\left(\psi_{k-1},\;\psi_{k}\right)$ can be denoted by *τ_k_* and its size by $\Delta_{k}=\psi_{k}-\psi_{k-1}$. Within the interval *τ_k_*, the periodic coefficient matrix ***C****_i_* can be replaced by a constant coefficient matrix ***C****_k_*. And ***A*** is the periodic coefficient matrix of the periodic system.

According to the Equations (20) and (21), the stable domain of the DSCR can be obtained. Usually, 60 < *N_k_* < 100, and J≥2 [40]. Therefore, the period *T* of Equation (20) is divided into 100 parts, and *J* = 4. The other parameters of the DSCR are listed in Table 2.

### 4.2. Three Stable Forms of the DSCR 

The modulus of the system characteristic multiplier varies with the control parameters of the DSCR, and corresponds to three typical motion states of asymptotically stable motion, periodic motion, and chaotic motion.

**Figure 6:**
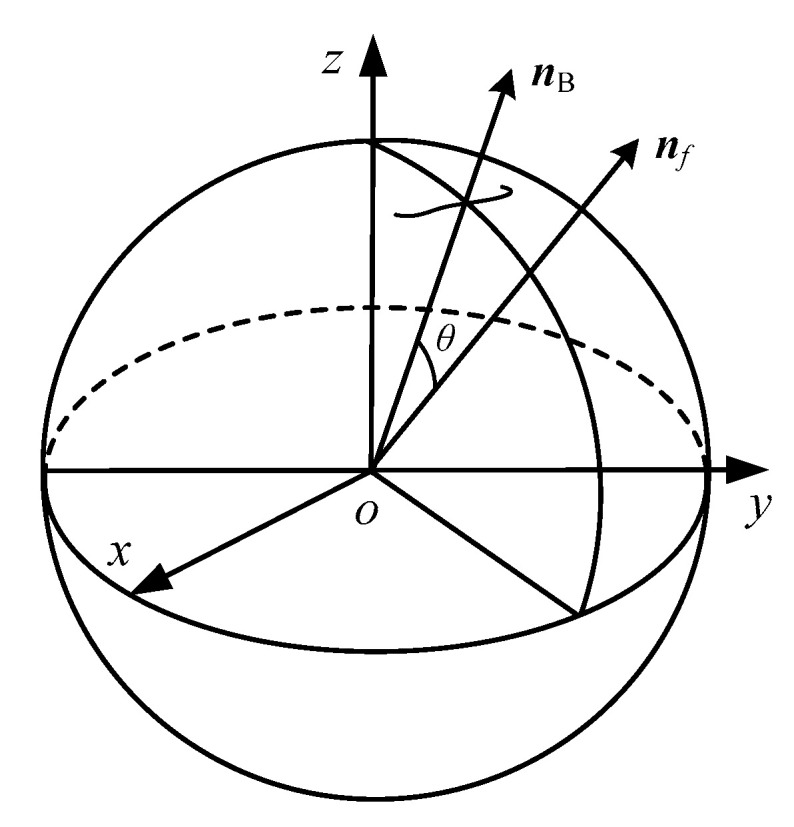
The orientation relationship between the polar axes ***n**_f_* and the target orientation ***n_B_***.

Since the polar axis ***n**_f_* should follow ***n_B_*** to change its orientation, then ***n_B_*** can be thought as the target orientation. When the DSCR is in different motion state, the polar axis ***n**_f_* and the target orientation ***n_B_*** have different orientation relations, the angle *θ* between ***n**_f_* and ***n_B_*** is defined as the orientation error of the system, as shown in Figure 6, and

(22) $$\theta =\arccos\left(n_{f}n_{B}/\left|n_{f}\right|\left|n_{B}\right|\right)$$

where ***n**_f_* and ***n_B_*** represents the orientation of the polar axis ***n**_f_* and the URMF in the fixed coordinate system *oxyz* respectively, and

(23) $$\left\{ \begin{array}{l} n_{f}={\left(\sin\alpha \cos\beta ,-\sin\beta ,\cos\alpha \cos\beta \right)}^{T}\\ n_{B}={\left(\sin\alpha_{1}\cos\beta_{1},-\sin\beta_{1},\cos\alpha_{1}\cos\beta_{1}\right)}^{T} \end{array}\right.$$

where *α* and *β* are the altitude angle and the azimuth angle of the polar axis ***n**_f_*. *α*_1_ and *β*_1_ are the altitude angle and the azimuth angle of the target orientation ***n_B_***.

#### 4.2.1. Asymptotically Stable Motion

**Figure 7:**
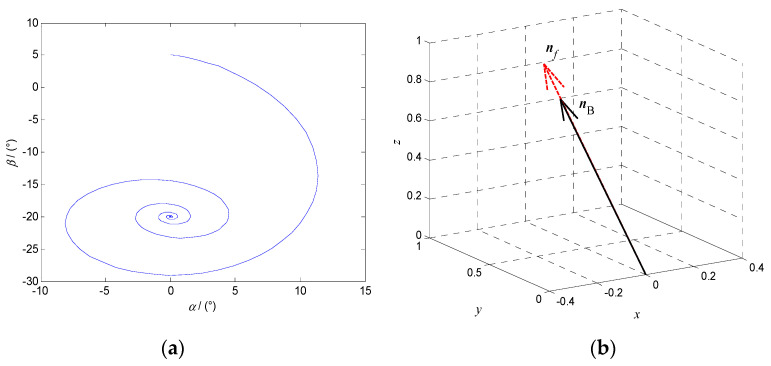
Asymptotically stable motion of the DSCR when *ω* = 18π rad/s, *B* = 7 mT, *l* = 0 mm. (**a**) The system phase diagram; (**b**) The orientation relationship between ***n**_f_* and ***n_B_***.

When the modulus of the characteristic multiplier is less than 1, the system phase diagram with the altitude angle *α* and the azimuth angle *β* as state variables is an asymptotically stable curve, as shown in Figure 7a. When the system reaches the steady state, the polar axis ***n**_f_* and the target orientation ***n_B_*** coincide in the fixed coordinate system *oxyz*, as shown in Figure 7b.

**Figure 8:**
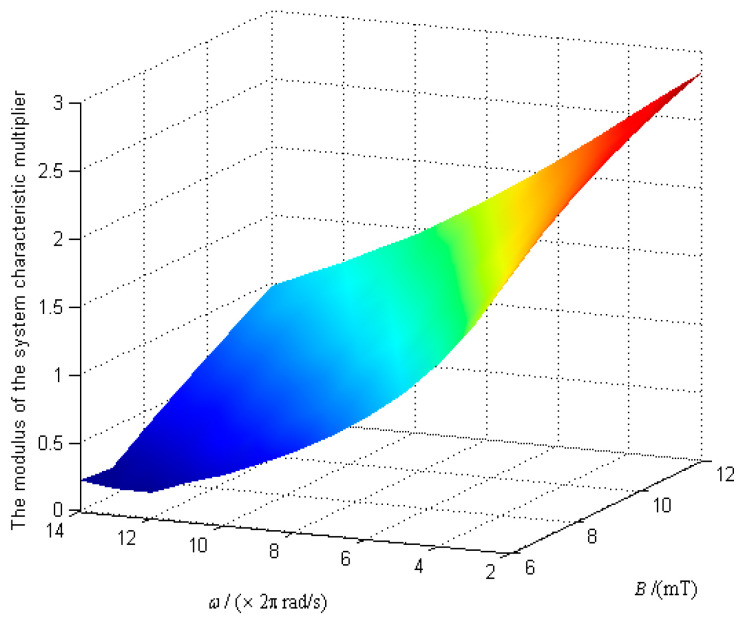
The modulus of the system characteristic multiplier varies with *ω* and *B*.

When the angular velocity of the URMF *ω* and the magnetic induction intensity *B* vary widely, the variation law of the modulus of the system characteristic multipliers was obtained, as shown in Figure 8.

**Figure 9:**
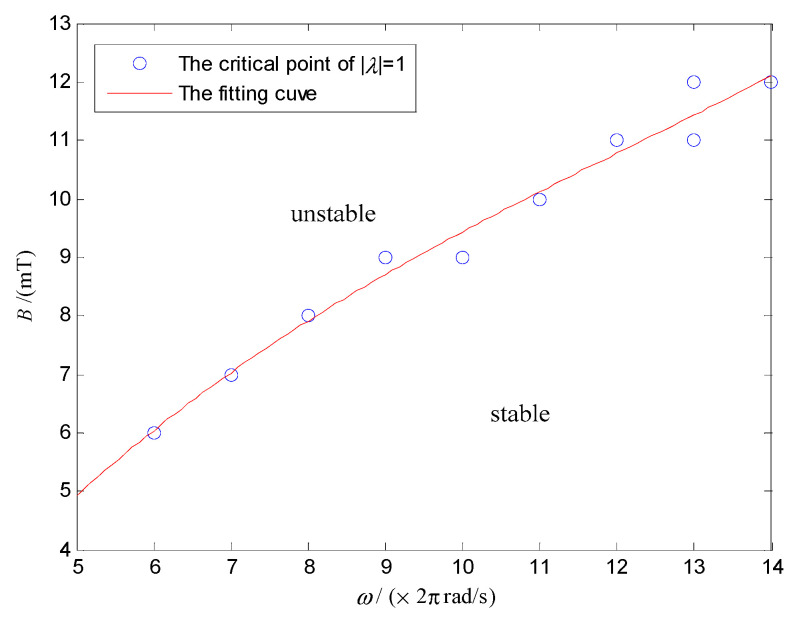
Asymptotically stable domain of the DSCR in the parameter space of *ω* and *B*.

Figure 8 shows that the modulus of the system characteristic multiplier decreases with *ω* and increases with *B*. The critical points of Figure 8, which satisfy the modulus of the system characteristic multipliers λ equal 1 was extracted, and the data was fitted by the least square method. Then, the stability domain of the system in the parameter space of *ω* and *B* can be obtained, as shown in Figure 9.

In Figure 9, the stability domain is divided into two parts by the critical point of |λ|=1. In the upper region, |λ|>1, and the system is unstable. On the contrary, the DSCR can keep the posture stable in the lower region of |λ|<1. Figure 9 shows that the posture stability of the DSCR can be improved by increasing the angular velocity of the URMF and decreasing the magnetic induction intensity. The reason is that when the rotational speed of the upper hemisphere increases with the URMF, the stability of the system can be improved under the gyroscopic effect. While the torque balance of the system may be destroyed by increasing the magnetic induction intensity.

#### 4.2.2. Stability of the Periodic Motion

**Figure 10:**
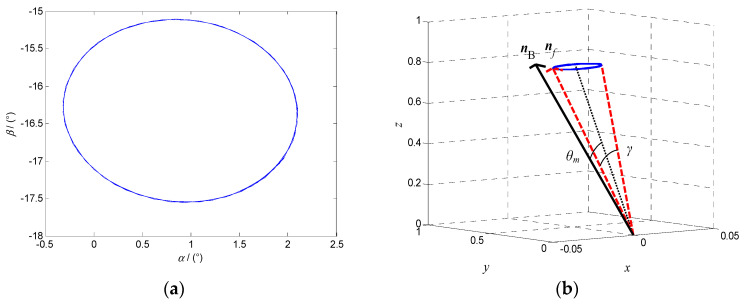
Periodic motion of the DSCR with *ω* = 18π rad/s, *B* = 7 mT, *l* = −2 mm. (**a**) The steady phase diagram of the system; (**b**) The precession of the polar axis ***n**_f_*.

When the modulus of the system characteristic multiplier is equal 1, the steady state phase diagram of the system with the altitude angle *α* and the azimuth angle *β* as state variables is a curve of periodic oscillation, as shown in Figure 10a, and the polar axis ***n**_f_* precesses near ***n*_B_**, as shown in Figure 10b.

**Figure 11:**
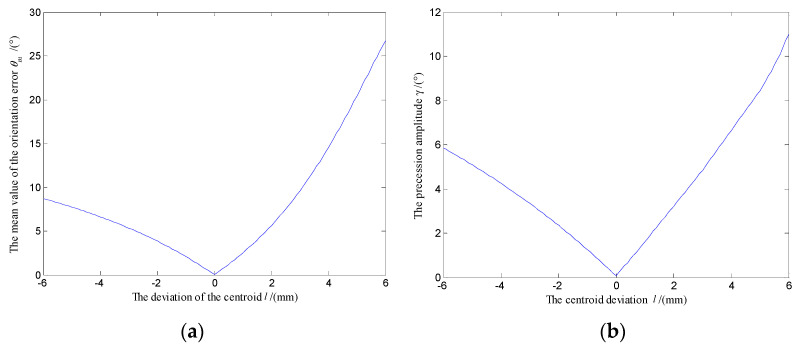
Variation law of the polar axis ***n**_f_* with the centroid deviation *l*. (**a**) Variation of orientation error *θ_m_* with centroid deviation *l*; (**b**) Variation of precession amplitude *γ* with centroid deviation *l*.

In order to explore the precession law of the polar axis ***n**_f_*, the angle *θ_m_* between the equilibrium position of the polar axis ***n**_f_* and the target orientation ***n_B_*** is defined as the mean orientation error of the system, and the swing angle *γ* of the polar axis ***n**_f_* is defined as the precession amplitude of the system, as shown in Figure 10b. Fix *ω* = 18π rad/s, *B* = 7 mT, the variation law of *θ_m_* and *γ* with the centroid deviation *l* were obtained, as shown in Figure 11a,b, respectively.

Figure 11 shows that when the centroid approaches the sphere center along the polar axis ***n**_f_* from below (*l* < 0), the mean orientation error and the precession amplitude of the system are both decreasing. When the centroid coincides with the sphere center (*l* = 0), the mean orientation error and the precession amplitude of the system are zeros. When the centroid deviates the sphere center along the polar axis ***n**_f_* from upwards (*l* > 0), the mean orientation error and the precession amplitude of the system both keep increasing. At the same time, compared with the upward deviation of the centroid along the polar axis ***n**_f_*, when the centroid is deviated downward, the mean orientation error and the precession amplitude of the system are smaller. Therefore, in the assembly and manufacturing process, the centroid of the DSCR should be coincident with the sphere center as far as possible.

**Figure 12:**
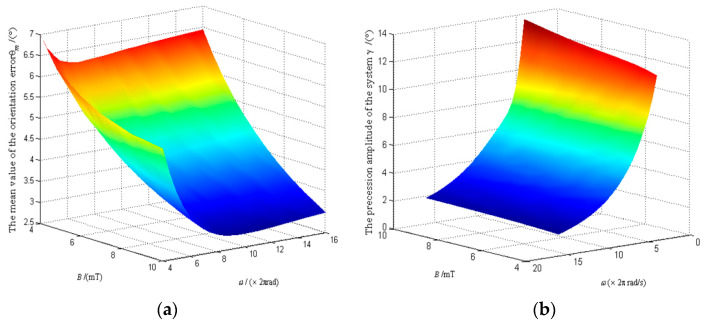
Variation law of the polar axis ***n**_f_* with *ω* and *B.* (**a**) Variation of orientation error *θ_m_* with *ω* and *B*; (**b**) Variation of precession amplitude *γ* with *ω* and *B*.

When the angular velocity of the UMMF *ω* and the magnetic induction intensity *B* vary over a wide range, the variation law of mean orientation error *θ_m_ and* the precession amplitude *γ* with *ω* and *B* as the control variables are showed in Figure 12a,b, respectively.

Figure 12a shows that the mean orientation error of the system can be reduced by increasing *ω* and *B* simultaneously. While Figure 12b shows that increasing *ω* and decreasing *B* can significantly reduce the precession amplitude of the system.

**Figure 13:**
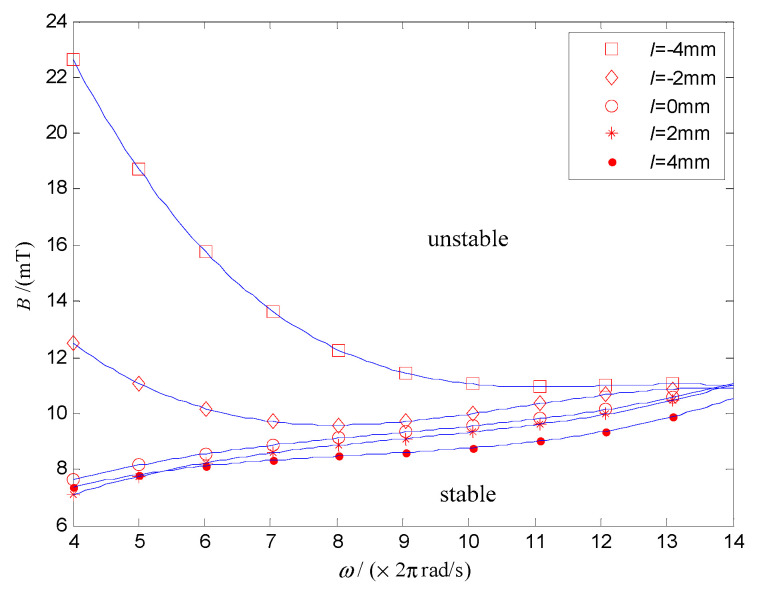
Stability domain of the DSCR for the periodic motion.

To explore the stability of the DSCR for the periodic motion, taking *ω* and *B* as the control parameters, the stability domain of the system under different centroid deviation is shown in Figure 13. The upper and lower areas of the curve represent the stable domain and unstable domain, respectively. Similar to Figure 9, Figure 13 shows that increasing the angular velocity of the URMF and decreasing the magnetic induction intensity can improve the stability of periodic motion of the system. 

#### 4.2.3. Chaotic Motion

**Figure 14:**
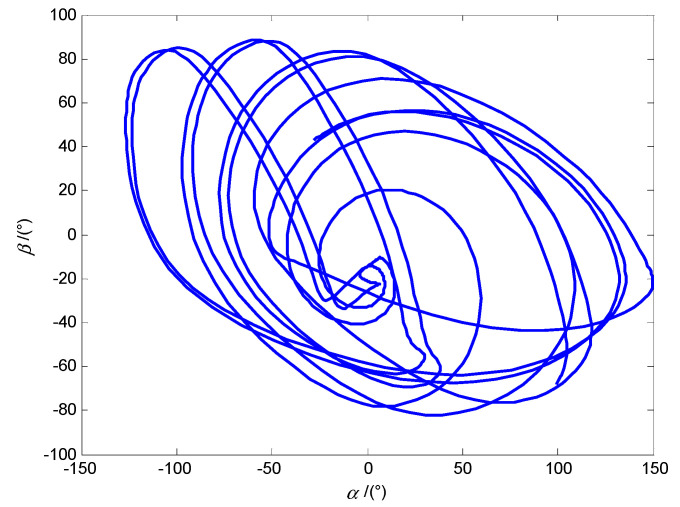
The system phase diagram of the DSCR for the chaotic motion.

As shown in Figure 14, when the modulus of the system characteristic multiplier is greater than 1, the system phase diagram with the altitude angle *α* and the azimuth angle *β* as state variables is chaotic, and the posture of the DSCR is unstable, which corresponding to the control condition of *ω* = 18π rad/s, *B* = 12 mT, *l* = 0 mm.

## 5. Experiment and Discussion

**Figure 15:**
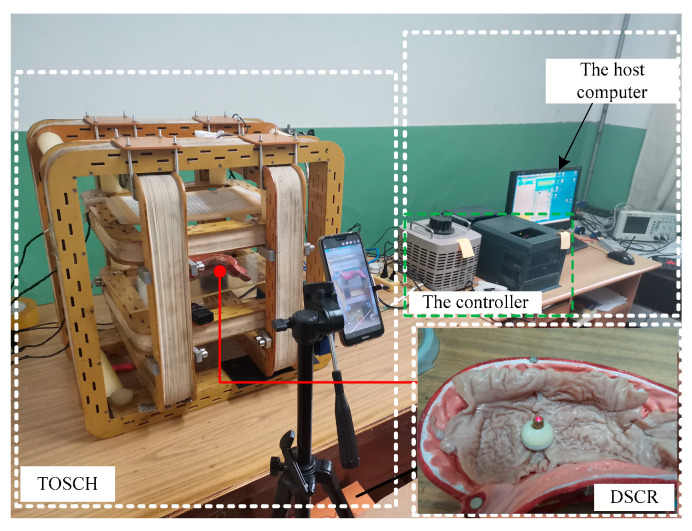
The experiment platform of the control system of the URMF.

To verify the theoretical analysis results, an experiment platform as shown in Figure 15 was built. The platform consists of the host computer, the controller, the TOSHC, and the DSCR. When the angular velocity of the UMMF *ω*, the magnetic induction intensity *B,* and the orientation of the URMF ***n_B_*** are input to the host computer, the controller can generate three-phase electric power that meet the control requirements, and the URMF can be generated after the three-phase alternating current are fed into the TOSHC. Since the orientation of the URMF can be controlled by the direction cosine of ***n_B_***, and the axis ***n**_f_* of the DSCR can follow ***n_B_*** to change its orientation, then the posture of the DSCR can be controlled by ***n_B_***. In order to simulate the environment of the GI tract, the isolated porcine intestinal tissue was spread on the surface of the stomach model.

### 5.1. Principle of the Polar Axis Orientation Measurement

**Figure 16:**
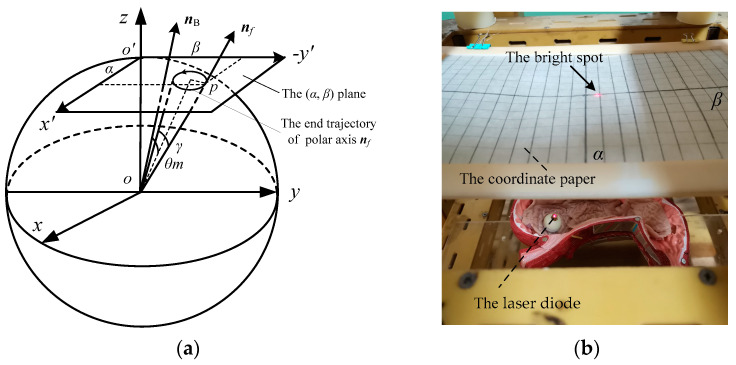
The orientation measuring device of the polar axis ***n**_f_*. (**a**) The schematic diagram; (**b**) The physical diagram.

The schematic diagram and physical diagram of the orientation measuring device of the polar axis ***n**_f_* are shown in Figure 16a,b, respectively. As shown in Figure 16a, the unit sphere with the DSCR spherical center *o* as the coordinate origin, and the unit sphere is intersected with the *oz* axis of the fixed coordinate system *oxyz* at the point *o*’. The tangent plane (*x*′, *y*′) of the unit sphere is parallel to the plane (*x*, *y*). According to Figure 3, the coordinates of the point *p*, which is the intersection point of the polar axis ***n**_f_* and the (*x*′, *y*′) plane can be obtained as

(24) $$x^{\prime }=\tan\alpha ,\;y^{\prime }=-\sec\alpha \tan\beta$$

When the polar axis ***n**_f_* moves in a small range near the *oz* axis, the second order small quantities of *α*, *β* are omitted, and the above equation can be simplified as

(25) $$x^{\prime }=\alpha ,\;y^{\prime }=-\beta$$

The *x*′ axis is called *α* axis, and the −*y*′ axis is called *β* axis, so the trajectory of the polar axis ***n**_f_* on the unit sphere can be approximately replaced by the trajectory on the (*α*, *β*) plane, that is, the plane pole trajectory. Moreover, the mean orientation error and the precession amplitude of the system are represent by the angles *θ_m_* and *γ*, respectively.

As shown in Figure 16b, the wireless image transmission module of the DSCR was replaced with a laser diode. The coordinate paper is placed at *h* = 100 mm above the sphere center of the DSCR. The bright spot of the laser diode on the coordinate paper can reflect the end motion trajectory of the polar axis ***n**_f_* in real time, and the trajectory can be recorded by the camera. The horizontal and vertical axes of the coordinate paper correspond to the altitude angle *α* and the azimuth angle *β*, respectively. Moreover, each scale on the coordinate paper represents 10°.

### 5.2. The Posture Stability Experiment

**Figure 17:**
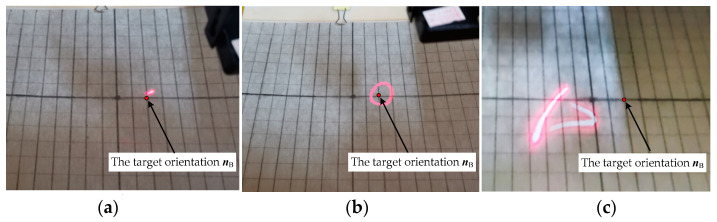
Snapshots of the end motion trajectories of polar axis ***n**_f_*. (**a**) Asymptotically stable motion; (**b**) Period motion; (**c**) Chaotic motion.

To verify the posture stability of the DSCR, three groups of cross experiments were designed as shown in Table 3, and the DSCR with no centroid deviation was used. Set the target orientation ***n_B_*** = (0°, −20°), when *ω* and *B* are controlled to change in turn, the end motion trajectories of the polar axis ***n**_f_* are shown in Figure 17a–c, respectively.

Figure 17a shows that the end motion trajectory of the polar axis ***n**_f_* is a fixed point when *ω* = 18π rad/s, *B* = 7 mT, which indicates that the system is asymptotically stable and the polar axis ***n**_f_* coincides with the target orientation ***n_B_***. While Figure 17b shows that the end motion trajectory of the polar axis ***n**_f_* is a curve of the periodic motion when *ω* = 14π rad/s, *B* = 7 mT, which indicates that the polar axis ***n**_f_***** makes the precession motion around the target orientation ***n_B_***. Moreover, the end motion trajectory of the polar axis ***n**_f_***** in Figure 17c is an irregular curve, indicating the chaotic motion of the system for the control parameters of *ω* = 18π rad/s, *B* = 12 mT. The above three groups of experiment results are consistent with the results in Figure 9.

### 5.3. The Precession Experiment

**Figure 18:**
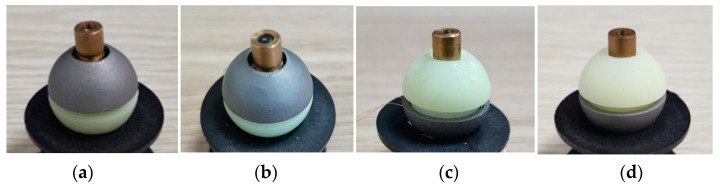
Four DSCR models with different centroid deviation. (**a**) *l* = 4 mm, (**b**) *l* = 2 mm, (**c**) *l* = −2 mm, (**d**) *l* = −4 mm.

To verify the precession characteristics of the polar axis ***n**_f_* when the DSCR makes the period motion, four DSCR models as shown in Figure 18 were 3D printed and assembled. The four DSCR models have the same weight and size except the centroid deviation, and the centroid deviation along the polar axis ***n**_f_* is 4 mm, 2 mm, −2 mm, −4 mm, respectively.

**Figure 19:**
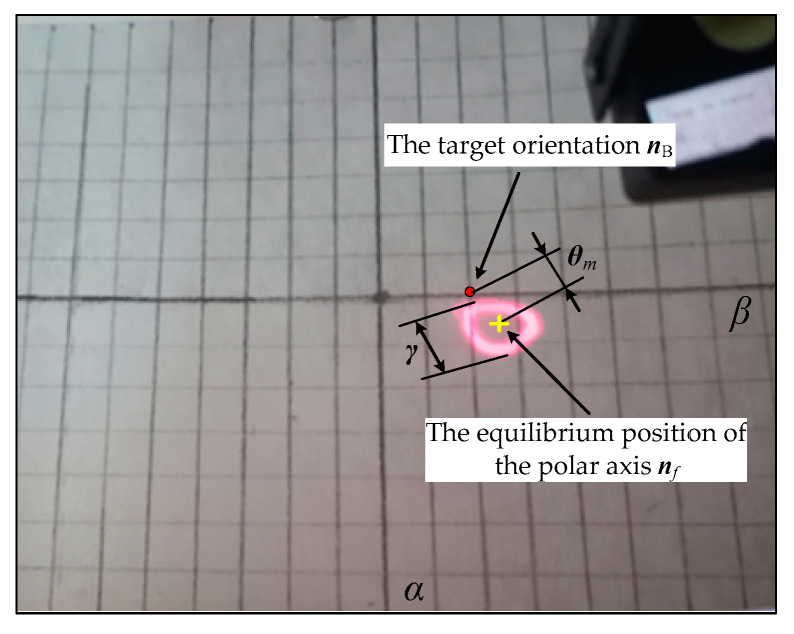
Snapshots of the end motion trajectories of polar axis ***n**_f_* of the centroid deviation *l* = −4 mm.

Set *ω* = 18π rad/s, *B* = 7 mT, and the target orientation ***n_B_*** = (0°, −20°), the end motion trajectories of the polar axis ***n**_f_* of the centroid deviation *l* = −4 mm is shown in Figure 19. From the equilibrium position and the end motion trajectory of the polar axis ***n**_f_*, the mean orientation error *θ_m_* and the precession amplitude *γ* as shown in Figure 16a can be obtained. Table 4 shows the precession results of the polar axis ***n**_f_* for the four DSCR models of Figure 18.

Table 4 shows that when the centroid approaches the sphere center from above or below, the mean orientation error and the precession amplitude of the system both gradually decreases. When the centroid is deviated up or down the same distance along the polar axis ***n**_f_*, the mean orientation error and the precession amplitude are smaller when the centroid moves down. The experiment results are consistent with the simulation results of Figure 11. The error of theoretical calculation and experimental data may be caused by the manufacturing and assembly errors of the DSCR and the system error of the orientation measuring device of the polar axis ***n**_f_*.

## 6. Conclusions

The DSCR with a dual-spin structure driven by the URMF was proposed, which can realize the fixed-point posture adjustment in the passive mode and the rolling locomotion in the active mode. The posture dynamics equation of the DSCR and the stability domain for the asymptotically stable motion and the periodic motion based on the Floquet–Lyapunov theory were obtained.

In general, we conclude that the DSCR makes the asymptotically stable motion, the periodic motion, the chaotic motion respectively, when the system characteristic multipliers less than 1, equal to 1, and greater than 1 are satisfied. In detail, increasing the angular velocity of the URMF and reducing the magnetic induction intensity can improve the posture stability of the DSCR. Decreasing the centroid deviation, increasing the angular velocity of the URMF can reduce the mean orientation error and the precession amplitude of the system. At the same time, compared with the upward deviation of the centroid along the polar axis, when the centroid is deviated downward, the orientation error and the precession amplitude of the system are smaller.

This research has laid a solid foundation for the structural improvement and the posture control of the DSCR.

## Figures and Tables

**Table 1:** The main structural parameters of the DSCR.

Name	Value/Material
The upper hemisphere shell	ABS Plastics
The sleeve	Aluminum alloy
The NdFeB permanent magnet	*Φ*7.5 × *Φ*6 × 5 mm
The wireless image transmission module (WITM)	-
The central axis	Aluminum alloy
The lower hemisphere shell	ABS Plastics
The bearing	*Φ*6 × *Φ*3 × 2.5 mm

**Table 2:** The control parameters of the DSCR.

Parameter	Value
The polar inertia moment of the upper hemisphere	*J*_1_ = 1.05 × 10^−7^ kg·m^2^
The polar inertia moment of the lower hemisphere	*J*_2_ = 7.34 × 10^−8^ kg·m^2^
The equatorial inertia moment of the DSCR	*J*_e_ = 1.97 × 10^−7^ kg·m^2^
The viscous damping coefficient	*k* = 1.65 × 10^−5^
Magnetic dipole moment of the NdFeB	*m* = 0.2 A·m^2^
The normal vector of the URMF	***n_B_*** = (0°, −20°)
The angular velocity of the URMF	*ω* = 18π rad/s
The magnetic induction intensity	*B* = 7 mT
The initial posture angles of the DSCR	(10°, 15°)
The slip angle *δ*	10°

**Table 3:** The cross experiments of the posture stability of the DSCR.

Experiment Number	The Angular Velocity of the URMF *ω*/(rad/s)	The Magnetic Induction Intensity *B*/(mT)	The Motion Law of the DSCR
(a)	18π	7	Asymptotically stable
(b)	14π	7	Period motion
(c)	18π	12	Chaotic motion

**Table 4:** The precession results of the DSCR for different centroid deviation.

Centroid Deviation /(mm)	The Mean Orientation Error *θ_m_*/(°)		The Precession Amplitude γ/(°)	
	Theoretical Value	Experiment Value	Theoretical Value	Experiment Value
4	14.5	19.5	6.7	15.9
2	5.6	11.1	5.6	13.4
−2	3.8	9.2	3.8	10.1
−4	6.6	13.3	4.5	12.6
